# Quantum Computing and the Future of Neurodegeneration and Mental Health Research

**DOI:** 10.3390/brainsci14010093

**Published:** 2024-01-18

**Authors:** George B. Stefano

**Affiliations:** Department of Psychiatry, First Faculty of Medicine, Charles University and General University Hospital in Prague, Ke Karlovu 11, 120 00 Prague, Czech Republic; gstefano@sunynri.org

## 1. Introduction

Quantum computing and supercomputing are two distinct approaches that can be used to solve complex computational problems. Supercomputers first became available for general use in the mid-1990s and are currently employed routinely to solve calculation-intensive problems, for example, weather forecasting and molecular modeling. Similar to standard desktop computers, supercomputers process data using bits (binary digits), which can be in an “on” or “off” state, represented by 0 or 1. However, while a simple computer typically has only one central processing unit (CPU), a supercomputer can have as many as 100,000 interconnected CPUs that process data in parallel [1]. By contrast, quantum computers process data using qubits (quantum bits) that can be in multiple states simultaneously and that permit calculations to be performed much more rapidly due to their potential for superposition (i.e., positional uncertainty) and entanglement (information exchange) [1]. Quantum computers can process extremely large datasets and provide solutions to complicated optimization problems at exponentially more rapid rates [2,3]. Furthermore, while supercomputers perform parallel processing using multiple cores/nodes working in tandem, quantum computers leverage quantum parallelism to explore multiple (in some cases, all) potential solutions simultaneously [2,3]. While supercomputers rely on established technology, routine use of quantum computers awaits further advances, for example, the development of superconducting qubits, topological qubits, trapped ions, and, most recently, dipolar interactions between molecules placed in optical tweezer arrays [4,5]. While yet to be routinely used, quantum computing has the potential to revolutionize many aspects of our daily lives as it can rapidly generate solutions to data-intensive, complex problems.

## 2. Neurodegeneration

Supercomputing has already made several important inroads into our understanding of neurodegenerative processes. For example, Rizo et al. [6] generated all-atom molecular dynamics simulations of membrane fusion events that lead to neurotransmitter release. Likewise, Moskal et al. [7] performed an artificial intelligence (AI)-guided screen that revealed the direct impact of a lipid-lowering drug on the survival of dopaminergic neurons, while Eickhoff et al. [8] used machine learning algorithms to examine brain aging in a cohort of patients diagnosed with Parkinson’s disease. Quantum computing has the potential to improve our understanding of neurodegenerative diseases and accelerate the development of novel treatment strategies. Because quantum computing can generate simulations of the behavior and interactions of individual molecules in complex biological systems, this will also serve as a means to accelerate drug discovery [9]. An understanding of the three-dimensional structure of proteins, including their capacity for folding and misfolding, will provide important molecular insights into the pathogenesis of neurodegenerative disorders, including Alzheimer’s and Parkinson’s diseases [10]. Quantum computing will also facilitate a more in-depth analysis of massive genomic and proteomic datasets and may uncover novel genetic polymorphisms, patterns, and biomarkers that can be used for early detection and/or tracking disease progression. Furthermore, this new technology may provide solutions to complex optimization problems and thus improve strategies used for individual treatment planning for patients diagnosed with neurodegenerative diseases. Among these, quantum computing algorithms may be used to determine personalized treatment plans and the most effective combination of drugs based on an individual’s genetic and medical profile [10,11]. As one example, Ugbaja et al. [12] reviewed current computational efforts, including several fledgling quantum approaches that have been used to explore the design and evaluation of beta-secretase inhibitors as a treatment for Alzheimer’s disease. However, quantum computing technology still faces numerous challenges, notably those involving error correction, scalability, and accessibility. Thus, its practical applications to neurodegeneration research and treatment may take some time to materialize.

## 3. Mental Health

Machine learning and AI have already had a substantial impact on mental health. For example, many AI “chatbots” currently used to provide mental health support were initially trained using natural language processing machine learning algorithms [13]. However, an improved overall understanding of psychiatric disease will require extensive and computation-intensive analyses of complex datasets. Thus, quantum computing has the potential to improve our overall understanding of mental health and uncover new and personalized treatment strategies [14,15]. As noted earlier, quantum computing algorithms may accelerate drug discovery because they can generate simulations of complex molecular events and interactions more rapidly and accurately than can be achieved using standard computing technology. These features will add critically to our efforts to improve the diagnosis of depression, schizophrenia, and bipolar disorder, and develop new, more effective, and potentially personalized medication strategies to treat these conditions. Quantum computing algorithms may be used to analyze an extensive array of genomic, proteomic, and neuroimaging data, and identify the biomarkers associated with specific psychiatric disorders. Importantly, quantum computers may be capable of simulating the human brain more accurately than can be achieved employing standard methods. This information will help us identify disordered neural circuits and network dynamics, thereby providing critical information and leading to the development of targeted interventions [14,15]. Finally, quantum computing will likely enhance and refine predictive modeling strategies currently used to determine mental health outcomes. Because they are capable of simultaneous analysis of genetic, behavioral, historical, clinical, and environmental data, quantum algorithms may provide insight into factors that increase the risk of developing mental health disorders, thereby facilitating early intervention.

## 4. Conclusions

Quantum computing is an evolving field that may ultimately revolutionize healthcare and medical research. Quantum computers are uniquely capable of processing vast amounts of data both quickly and efficiently. Thus, quantum computing algorithms can be used to identify disease-specific biomarkers to facilitate early detection, ongoing monitoring, and the development of targeted and potentially personalized therapeutic strategies for individuals with neurodegenerative and psychiatric diseases. These tools may also be used to identify existing drugs that can be repurposed for new medical applications and improve the design of clinical trials. Specifically, researchers can use quantum algorithms to optimize trial parameters, patient cohorts, and treatment protocols to create more efficient and effective clinical trials. Furthermore, this technology can be used to improve the processing and analysis of results from magnetic resonance imaging, computed tomography, and positron emission tomography, which will permit clinicians to provide more accurate disease diagnoses. In addition to clinical research, quantum computing may be used to optimize healthcare logistics and resource allocation, including hospital operations, patient scheduling, and supply chain management. Accurate assessments of these parameters will enhance both the access to and cost-effectiveness of healthcare. However, some means will need to be developed to ensure the security of sensitive personal medical data. As quantum computing matures, we will develop an even greater appreciation of its potential to transform medicine and healthcare technologies. 
